# Clinical and Radiographic Outcomes Following Unintentional Extrusion of Cold Ceramic: A Case Series of Three Patients

**DOI:** 10.1002/ccr3.73569

**Published:** 2026-09-23

**Authors:** Jalil Modaresi, Maaz Anwer Memon, Mojtaba Mehrabanian

**Affiliations:** ^1^ Department of Endodontics, School of Dentistry Shahid Sadoughi University of Medical Sciences Yazd Iran; ^2^ Department of Oral Pathology, Shifa College of Dentistry Shifa Tameer‐e‐Millat University Islamabad Pakistan; ^3^ Institute of Dentistry, School of Medicine, Medical Sciences and Nutrition University of Aberdeen Aberdeen UK

**Keywords:** apical extrusion, bioceramic, calcium silicate cement, cold ceramic, nonsurgical root canal retreatment, periapical lesion

## Abstract

In three patients with unintentional extrusion of cold ceramic beyond the root apex, favorable clinical and radiographic outcomes were observed over 2–4 years. These descriptive observations do not establish the safety or biological effects of extruded material, but support careful clinical and radiographic follow‐up when patients remain asymptomatic.

## 1 Introduction

Successful root canal treatment depends on effective chemomechanical debridement of the root canal system, followed by three‐dimensional obturation that seals the prepared canal space and limits reinfection [1]. Ideally, obturation materials should remain confined within the root canal system; however, inadvertent extrusion beyond the apical foramen may occur, particularly in teeth with apical root resorption, immature apices, perforations, or extensive periapical destruction [2, 3]. The biological response to extruded filling materials depends largely on their physicochemical properties, tissue compatibility, and the extent and location of extrusion [4].

Conventional root canal obturation has traditionally relied on gutta‐percha used in conjunction with an endodontic sealer [5]. Although gutta‐percha remains widely used because of its favorable handling characteristics and long‐term clinical success, it is biologically inert, lacks adhesive properties, and does not stimulate hard tissue formation [6]. Extrusion of gutta‐percha and certain conventional filling materials beyond the apical foramen has been associated with persistent inflammation, foreign body reactions, delayed healing, and occasional postoperative discomfort [7]. Consequently, preservation of the apical constriction and accurate control of working length remain fundamental principles of endodontic treatment.

Calcium silicate‐based biomaterials are used for apical barrier formation, perforation repair, root‐end filling, regenerative procedures, and orthograde obturation because of their sealing ability, dimensional stability, biocompatibility, and bioactivity [8, 9, 10].

The cold ceramic (CC) was first introduced in 2000 as a calcium silicate‐based, mineral trioxide aggregate (MTA)‐like cement for endodontic applications. The principal component of CC is calcium hydroxide, and the material sets in the presence of moisture with an initial setting time of approximately 15 min and complete setting within 24 h [11]. Previous studies have reported sealing ability and biocompatibility of CC under selected experimental conditions [12, 13]. Clinical use of CC has also been described in challenging endodontic situations, including obturation of partially calcified root canals, although the available evidence remains limited to case‐based observations [14]. However, these findings cannot be directly extrapolated to the clinical behavior of the material unintentionally extruded beyond the root apex, and evidence regarding long‐term clinical outcomes in this setting remains limited.

This case series presents the clinical and radiographic outcomes of three patients in whom CC was unintentionally extruded into the periapical tissues during orthograde root canal treatment or retreatment with 2–4 years of follow‐up. The observations are presented descriptively without attributing the outcomes to the safety or biological effects of the extruded material.

## 2 Case History/Examination

Three patients, two females and one male aged 20–54 years, were included in this case series.

### 2.1 Case 1

A 20‐year‐old female presented with a previously treated mandibular left central incisor (#24), which had undergone nonsurgical root canal treatment 6 years earlier and had subsequently been restored with composite resin. Clinical examination revealed localized buccal swelling, tenderness to percussion, Grade I mobility, and periodontal indices, including probing depths, within normal limits. The medical history was non‐contributory.

### 2.2 Case 2

A 20‐year‐old male presented with pain and localized swelling involving the left mandibular anterior region associated with the mandibular left lateral incisor (#23) and mandibular left central incisor (#24). Clinical examination revealed fluctuant labial swelling adjacent to the mandibular incisors and tenderness to percussion. Periodontal indices, including probing depths, were within normal limits. The medical history was non‐contributory.

### 2.3 Case 3

A 54‐year‐old female presented with a previously treated maxillary right lateral incisor (#7), which had undergone root canal treatment 4 years earlier. She reported pain, tenderness to percussion, and recurrent drainage through a buccal sinus tract. Clinical examination revealed Grade I mobility, while periodontal indices, including probing depths, were within normal limits.

## 3 Methods: Differential Diagnosis, Investigations and Treatment

### 3.1 Case 1

**FIGURE 1:**
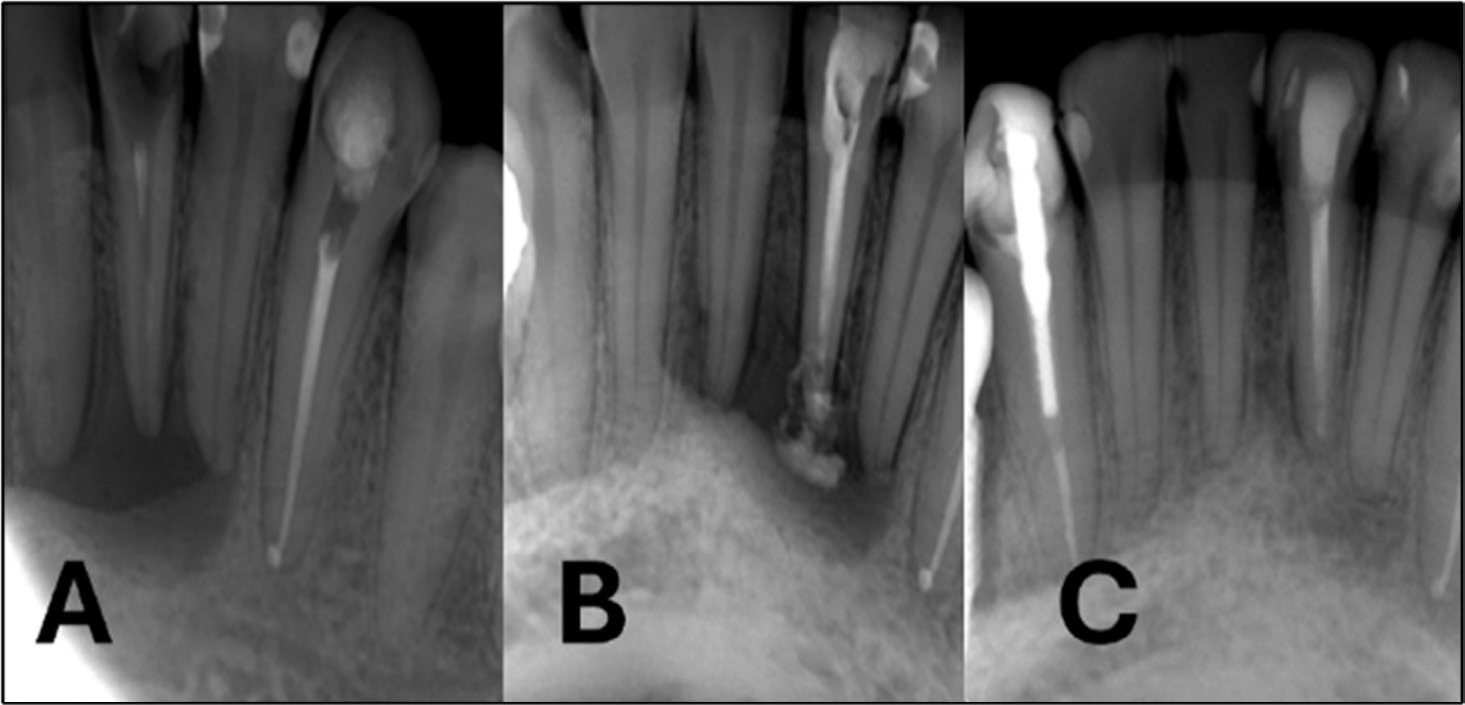
Case 1. (A) Preoperative periapical radiograph showing inadequate previous root canal treatment and a large periapical radiolucency associated with tooth #24. (B) Immediate postoperative radiograph following nonsurgical retreatment using a 5‐mm CC apical plug with apical extrusion of CC. (C) Four‐year follow‐up periapical radiograph showing that the previously observed periapical radiolucency was no longer evident and that the extruded CC was no longer distinguishable radiographically.

Radiographic examination showed inadequate previous root canal treatment characterized by poor‐quality obturation with multiple voids and a large periapical radiolucency exceeding 1 cm in diameter. The lesion extended from the distal aspect of tooth #23 to the mesial aspect of tooth #26 (Figure 1A). Adjacent teeth responded normally to both cold testing and electrical pulp testing. Based on the clinical and radiographic findings, tooth #24 was diagnosed as previously treated with an acute apical abscess.

Following local anesthesia and rubber dam isolation, the previous restoration was removed, and root canal retreatment was initiated. Existing filling materials were removed using Gates‐Glidden drills, Hedström files, and rotary instruments. Working length was established using an electronic apex locator (Root ZX; Morita, Tokyo, Japan) and confirmed radiographically. Apical patency was confirmed with a size 10 K‐file.

The canal was prepared using hand K‐files to size 35 followed by ProTaper rotary instrumentation to F2. Irrigation was performed with 5.25% sodium hypochlorite throughout instrumentation, followed by a final rinse with sterile 0.9% normal saline. After drying the canal with sterile paper points, CC was prepared by gradually incorporating the manufacturer's supplied liquid into the powder and spatulating for approximately 15–30 s until a homogeneous, workable consistency was achieved, in accordance with the manufacturer's instructions. The material was delivered using an MTA carrier. A 5‐mm apical plug was placed and vertically compacted using endodontic pluggers and spreaders. The remaining canal space was obturated with gutta‐percha (DiaDent) and Rickert sealer (PD).

Extrusion of CC beyond the apical foramen occurred during placement (Figure 1B). Given the extensive periapical lesion, loss of the natural apical constriction or inflammatory apical resorption may have contributed; however, the precise cause could not be established retrospectively.

### 3.2 Case 2

**FIGURE 2:**
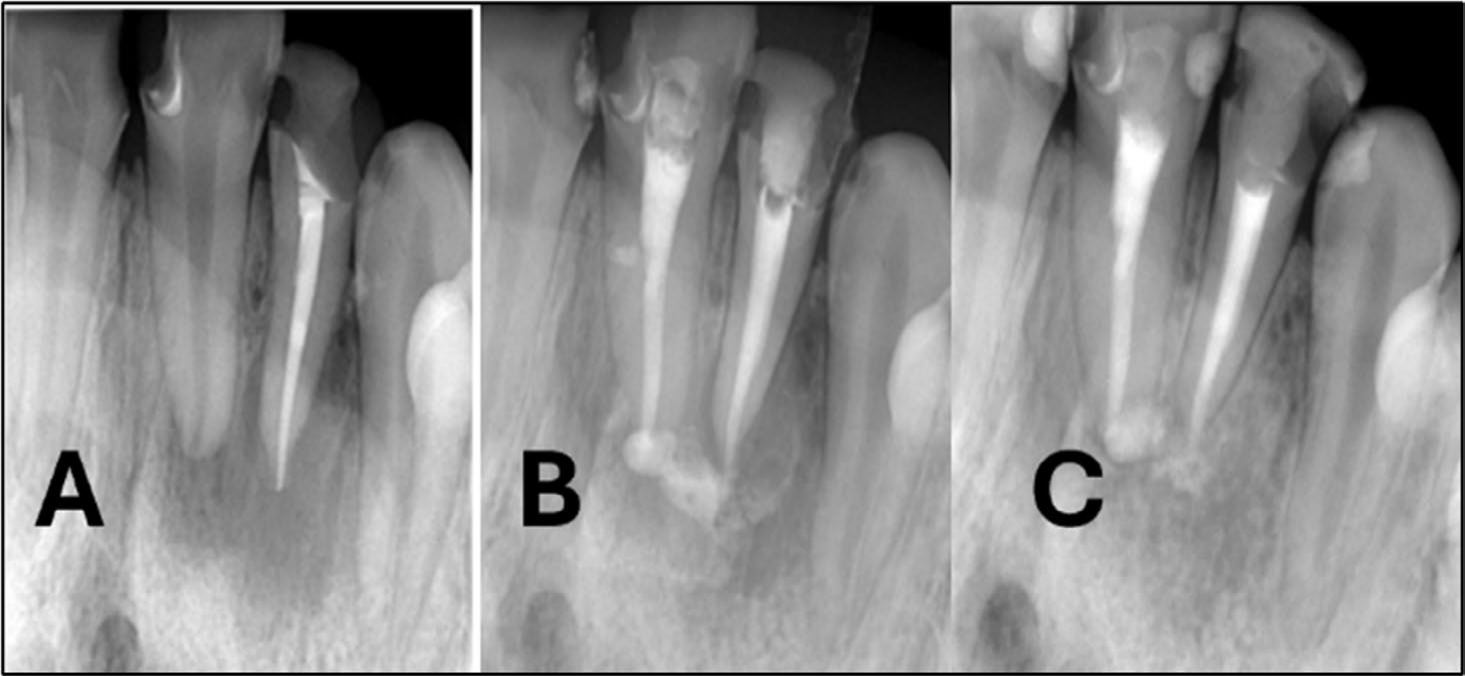
Case 2. (A) Preoperative periapical radiograph demonstrating a large periapical radiolucency involving teeth #23 and #24. (B) Immediate postoperative radiograph following placement of 5‐mm CC apical plugs, with extrusion of CC. (C) Two‐year follow‐up periapical radiograph showing that the previously observed periapical radiolucency was no longer evident and that the radiographic visibility of the extruded CC was reduced compared with the immediate postoperative image.

Radiographic examination showed a large periapical radiolucency involving the mandibular left central and lateral incisors (teeth #24 and #23) (Figure 2A). Tooth #24 had not previously undergone root canal treatment and was considered to require primary treatment, whereas tooth #23 had undergone nonsurgical root canal treatment 4 years previously and required retreatment. Based on the clinical and radiographic findings, tooth #24 was diagnosed as pulp necrosis with acute apical abscess, and tooth #23 was diagnosed as previously treated with acute apical abscess.

After administration of local anesthesia and rubber dam isolation, tooth #24 was accessed and working length was determined using an electronic apex locator and confirmed radiographically. Apical patency was confirmed during canal preparation with a size 10 K‐file. Chemomechanical preparation was performed using hand K‐files to size 40 and ProTaper rotary instrumentation to F2 with 5.25% sodium hypochlorite irrigation followed by normal saline. Following canal drying, a 5‐mm CC apical plug was prepared and delivered using the protocol described for Case 1. The remaining canal space was obturated with gutta‐percha and Rickert sealer.

At the subsequent appointment, retreatment of tooth #23 was performed. The existing root canal filling materials were removed using Gates‐Glidden drills, Hedström files, and chloroform. Working length was established using an electronic apex locator and confirmed radiographically, and apical patency was confirmed with a size 10 K‐file. The canal was prepared using the same instrumentation and irrigation protocol. CC was prepared and delivered using the same protocol described for tooth #24, and a 5‐mm apical plug was placed before the remaining canal space was obturated with gutta‐percha and Rickert sealer.

Extrusion of CC beyond the apical foramina occurred during placement (Figure 2B). Given the extensive periapical lesion, loss of the natural apical constrictions or inflammatory apical resorption may have contributed; however, the precise cause could not be established retrospectively.

### 3.3 Case 3

**FIGURE 3:**
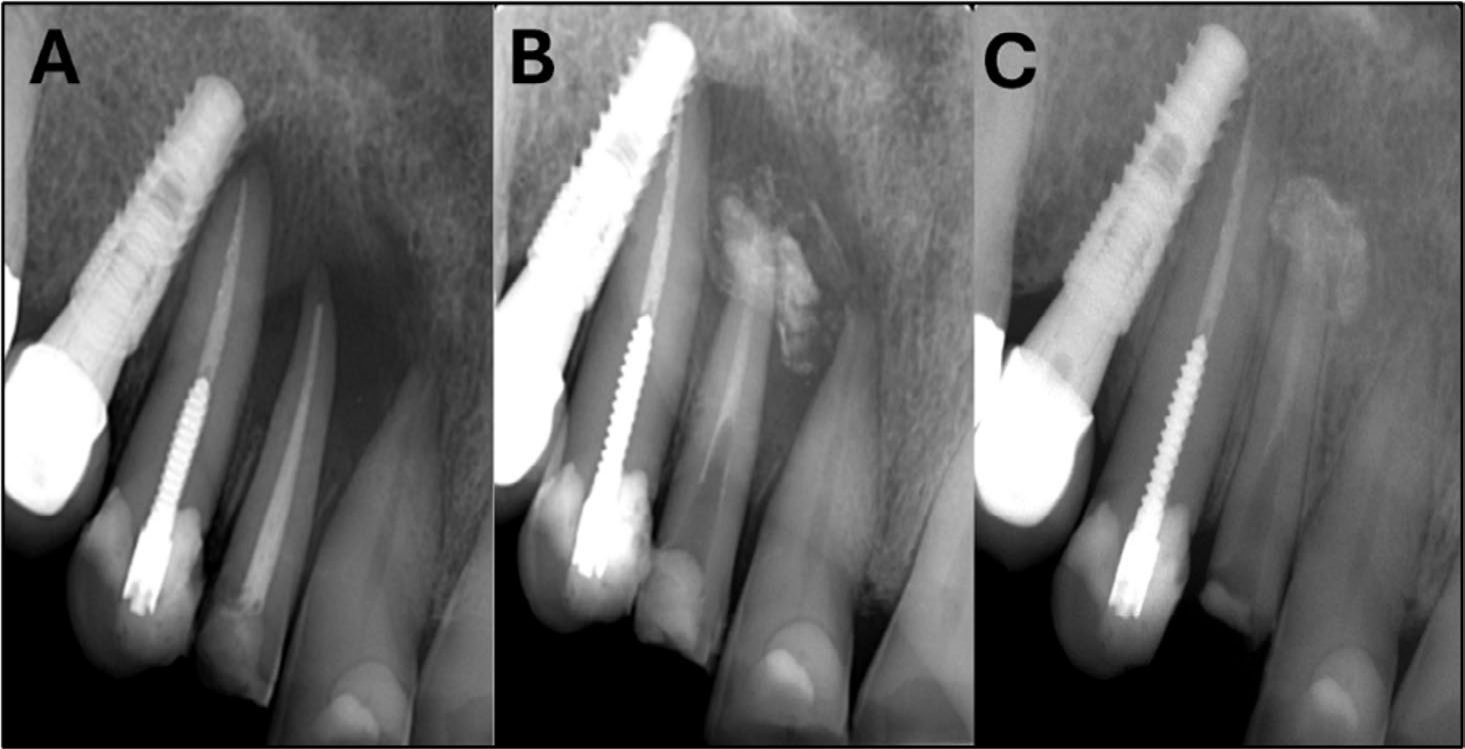
Case 3. (A) Preoperative periapical radiograph demonstrating a large periapical radiolucency associated with previously treated tooth #7. (B) Immediate postoperative radiograph following placement of a 5‐mm CC apical plug, with extrusion of CC beyond the root apex. (C) Two‐year follow‐up radiograph demonstrating that the previously observed periapical radiolucency was no longer evident.

Radiographic examination demonstrated a large periapical radiolucency associated with tooth #7 (Figure 3A). The adjacent central incisor (tooth #8) responded positively to pulp sensibility testing.

Based on the clinical and radiographic findings, #7 was diagnosed as previously treated with a chronic apical abscess.

Following administration of local anesthesia and rubber dam isolation, access was established, the existing root canal filling material was removed, the working length was determined using an electronic apex locator and confirmed radiographically, and apical patency was confirmed with a size 10 K‐file. Chemomechanical preparation was completed using ProTaper instrumentation to size F3 with copious irrigation using 5.25% sodium hypochlorite.

Persistent intracanal exudation prevented immediate obturation. Therefore, calcium hydroxide (PD, Vevey, Switzerland) was placed as an intracanal medicament for 2 weeks and the access cavity was sealed temporarily.

At the second appointment, the patient was asymptomatic. Calcium hydroxide was removed using normal saline irrigation, and the canal was dried with sterile paper points. A 5‐mm CC apical plug was prepared and delivered using the protocol described for Case 1, then vertically compacted before completion of obturation.

Extrusion of CC beyond the root apex occurred during placement (Figure 3B). Loss of the natural apical constriction or inflammatory apical resorption may have contributed; however, the precise cause could not be established retrospectively.

## 4 Outcome and Follow‐Up

### 4.1 Case 1

The patient remained asymptomatic throughout the follow‐up period. At the 4‐year review, clinical examination revealed no pain, swelling, sinus tract, and tenderness to percussion, together with stable periodontal findings. Radiographic assessment showed that the previously observed periapical radiolucency was no longer evident, and the extruded CC was no longer distinguishable radiographically (Figure 1C).

### 4.2 Case 2

At the two‐year follow‐up, the patient was asymptomatic, with no pain, swelling, or other clinical signs of persistent disease, and periodontal findings remained within normal limits. The previously observed periapical radiolucency was no longer evident on the follow‐up periapical radiograph (Figure 2C). The extruded CC was less radiographically distinguishable than on the immediate postoperative image.

### 4.3 Case 3

Two years later, the patient was referred back by her restorative dentist after removal of the existing post, core, and crown to determine whether further endodontic intervention was required. Clinical examination revealed no pain, swelling, sinus tract, or tenderness to percussion, and periodontal findings remained within normal limits. The previously observed periapical radiolucency was no longer evident on the follow‐up periapical radiograph (Figure 3C). Consequently, no additional endodontic treatment was indicated.

## 5 Discussion

Accidental extrusion of root canal filling materials beyond the apical foramen is generally considered undesirable because it may delay periapical healing and provoke inflammatory or foreign body reactions, particularly when materials with limited biocompatibility are displaced into the periapical tissues [15]. Nevertheless, the biological response to overextended filling materials depends largely on their physicochemical characteristics, biocompatibility, and interaction with the surrounding tissues [16]. In the present case series, all three patients with unintentional extrusion of CC were clinically asymptomatic at follow‐up, and the previously observed periapical radiolucencies were no longer evident on conventional periapical radiographs after 2–4 years. These observations are descriptive and cannot establish a causal relationship between CC extrusion and the observed outcomes.

Successful endodontic treatment relies on effective elimination of intracanal microorganisms followed by three‐dimensional sealing of the root canal system [17]. Ideally, obturation materials should terminate at or slightly short of the apical constriction. Previous studies have reported that overextension of conventional filling materials, particularly gutta‐percha and certain sealers, may be associated with lower healing rates and persistent periapical inflammation [18]. Histological investigations have also reported chronic inflammatory reactions and foreign body responses surrounding extruded gutta‐percha [19]. Consequently, clinicians generally attempt to avoid apical extrusion during obturation.

Calcium silicate‐based materials have been investigated because of their biocompatibility and sealing properties [20]. CC is an MTA‐like calcium silicate cement that has been introduced for multiple endodontic applications, including root‐end filling, perforation repair, apexification, and orthograde obturation [13, 14, 21]. An in vitro study on dental pulp cells and periodontal ligament fibroblasts has demonstrated that CC possesses favorable sealing ability, dimensional stability, and biocompatibility comparable to those of MTA [22]. Furthermore, the material releases calcium ions during hydration, which may contribute to hydroxyapatite formation and enhance biological sealing at the dentin‐material interface. These physicochemical properties may explain the favorable tissue response observed in the present cases. However, findings from laboratory studies and isolated clinical reports cannot be directly extrapolated to the behavior of CC when unintentionally extruded into human periapical tissues. Clinical evidence specifically addressing unintentionally extruded CC remains limited.

In Cases 1 and 2, the radiographic visibility of the extruded CC decreased during follow‐up, while the previously observed periapical radiolucencies were no longer evident. These findings should be interpreted cautiously because conventional periapical radiography cannot determine whether the apparent change reflected material resorption, tissue replacement, or another radiographic phenomenon. Clinical evidence from retrospective controlled studies of MTA apexification has shown that periapical healing can occur despite unintentional extrusion of MTA, although healing may be delayed compared with cases without extrusion [23]. However, these findings relate specifically to MTA and cannot be directly extrapolated to CC.

Another clinically important observation was the absence of persistent postoperative symptoms in all three patients. None of the cases demonstrated prolonged pain, swelling, sinus tract recurrence, or tenderness during the follow‐up period. Although the biocompatibility reported for calcium silicate‐based materials may represent one possible explanation for the absence of adverse tissue responses, the present case series cannot determine whether this contributed to the observed outcomes. Other components of treatment, including chemomechanical preparation, sodium hypochlorite irrigation, apical sealing, and in Case 3, intracanal calcium hydroxide medication, may also have contributed. Therefore, these observations do not establish the safety of extruded CC or support intentional extrusion. Clinical and radiographic follow‐up remains important when inadvertent extrusion has occurred, particularly to identify persistent symptoms or progressive radiographic abnormalities. Persistent pain, swelling, neurological disturbance, sinus tract recurrence, progressive radiographic abnormalities, or extrusion near anatomically sensitive structures should prompt further assessment and possible intervention.

Case 3 illustrates the use of CC in a challenging endodontic situation in which persistent intracanal exudation prevented immediate obturation following chemomechanical preparation. Short‐term intracanal medication with calcium hydroxide was therefore used before definitive obturation. CC is a hydraulic calcium silicate‐based material that sets in the presence of moisture, which may be clinically relevant when complete canal dryness is difficult to achieve [12, 24]. However, the favorable outcome in this case cannot be attributed specifically to CC, because chemomechanical preparation, sodium hypochlorite irrigation, intracanal calcium hydroxide medication, and subsequent obturation may all have contributed.

The favorable outcomes observed in this case series should not be interpreted as encouraging intentional overextension of obturation materials. Preservation of the apical constriction and confinement of filling materials within the root canal system remain fundamental principles of endodontic treatment. In the present cases, extrusion occurred unintentionally because of extensive inflammatory apical resorption and loss of the natural apical constriction. Therefore, accurate working‐length determination, apical gauging, controlled material delivery, and radiographic verification remain essential. Nevertheless, the present observations suggest that favorable clinical and radiographic outcomes can occur despite inadvertent CC extrusion, but they do not establish that extrusion is harmless or that CC itself contributed to healing.

## 6 Limitations

This case series has several limitations. First, only three cases were included, and there was no comparison group, preventing causal conclusions regarding the safety, biological behavior, or clinical effects of extruded CC. The favorable outcomes may have reflected chemomechanical preparation, irrigation, intracanal medication where used, apical sealing, and other treatment‐related factors rather than any specific biological effect of CC. Second, assessment of healing was based on clinical examination and conventional periapical radiography, without three‐dimensional imaging or histological evaluation. Consequently, the biological fate of the extruded material and the nature of the tissues occupying the previously radiolucent areas could not be determined. Third, the amount of extruded CC was not quantified, preventing assessment of any relationship between the extent of extrusion and outcome. Fourth, treatment protocols were not identical across the three cases; notably, Case 3 received calcium hydroxide intracanal medication for 2 weeks before definitive obturation. Finally, one author is the inventor and owner of cold ceramic and may have a commercial interest in its use, representing a potential source of interpretative bias. Independent studies with larger samples, standardized treatment protocols, quantitative assessment of extrusion, and appropriate three‐dimensional imaging are required to further evaluate the clinical behavior of unintentionally extruded CC.

## 7 Conclusion

In this three‐patient case series, favorable clinical and radiographic findings were observed over 2–4 years following unintentional extrusion of CC beyond the root apex. At follow‐up, the patients were asymptomatic, and the previously observed periapical radiolucencies were no longer evident on conventional periapical radiographs. The radiographic visibility of the extruded material decreased in some cases; however, its biological fate could not be determined. These observations are descriptive and do not establish the safety, efficacy, or biological behavior of extruded CC, nor can the outcomes be attributed specifically to the material. Careful clinical and radiographic follow‐up may be appropriate when inadvertent extrusion occurs, while accurate working‐length determination and controlled material placement remain essential to minimize the risk of extrusion.

## Author Contributions

**Jalil Modaresi:** investigation; resources; data curation; visualization, writing – review and editing. **Maaz Anwer Memon:** validation; writing – review and editing. **Mojtaba Mehrabanian:** conceptualization; data curation; visualization; writing – original draft; writing – review and editing; project administration; supervision. All authors approved the final version of the manuscript and agreed to be accountable for all aspects of the work.

## Funding

The authors have nothing to report.

## Ethics Statement

This case series was approved by the Research Ethics Committee of Shahid Sadoughi University of Medical Sciences, Yazd, Iran (approval code: IR.SSU.REC.1400.170).

## Consent

Written informed consent was obtained from all patients for publication of this case series and the accompanying clinical and radiographic images.

## Conflicts of Interest

Jalil Modaresi is the inventor and owner of cold ceramic and may have a financial interest in its commercial use. Mojtaba Mehrabanian has previously co‐authored a separate case report involving cold ceramic but has no financial or proprietary interest in the material. Maaz Anwer Memon declares no conflicts of interest.

## Data Availability

The clinical data supporting this case series are not publicly available because of patient confidentiality. De‐identified data may be available from the corresponding author upon reasonable request and subject to institutional and ethical requirements.

## References

[1] A. Gusiyska and E. Dyulgerova , “Clinical Approaches to the Three‐Dimensional Endodontic Obturation Protocol for Teeth With Periapical Bone Lesions,” Applied Sciences 13, no. 17 (2023): 9755.

[2] E. Rosen , T. Goldberger , S. Taschieri , M. Del Fabbro , S. Corbella , and I. Tsesis , “The Prognosis of Altered Sensation After Extrusion of Root Canal Filling Materials: A Systematic Review of the Literature,” Journal of Endodontics 42, no. 6 (2016): 873–879, 10.1016/j.joen.2016.03.018. 27133502

[3] J. Tanalp and T. Güngör , “Apical Extrusion of Debris: A Literature Review of an Inherent Occurrence During Root Canal Treatment,” International Endodontic Journal 47, no. 3 (2014): 211–221. 10.1111/iej.12137 23711187

[4] P. Saxena , S. K. Gupta , and V. Newaskar , “Biocompatibility of Root‐End Filling Materials: Recent Update,” Restorative Dentistry and Endodontics 38, no. 3 (2013): 119–127, 10.5395/rde.2013.38.3.119. PMC3761119 24010077

[5] L. Peng , L. Ye , H. Tan , and X. Zhou , “Outcome of Root Canal Obturation by Warm Gutta‐Percha Versus Cold Lateral Condensation: A Meta‐Analysis,” Journal of Endodontics 33, no. 2 (2007): 106–109, 10.1016/j.joen.2006.09.010. 17258624

[6] J. Dobrzańska , L. B. Dobrzański , L. A. Dobrzański , K. Gołombek , and A. D. Dobrzańska‐Danikiewicz , “Is Gutta‐Percha Still the “Gold Standard” Among Filling Materials in Endodontic Treatment?,” Processes 9, no. 8 (2021): 1467, 10.3390/pr9081467.

[7] M. Fernandes , “Nonsurgical Management of a Large Periapical Lesion Using Aspiration in Combination With a Triple Antibiotic Paste and Calcium Hydroxide,” Iranian Endodontic Journal 5, no. 4 (2010): 179–182. PMC4004795 24790632

[8] J. Camilleri , F. E. Montesin , K. Brady , R. Sweeney , R. V. Curtis , and T. R. P. Ford , “The Constitution of Mineral Trioxide Aggregate,” Dental Materials 21, no. 4 (2005): 297–303. 10.1016/j.dental.2004.05.010 15766576

[9] S.‐J. Lee , M. Monsef , and M. Torabinejad , “Sealing Ability of a Mineral Trioxide Aggregate for Repair of Lateral Root Perforations,” Journal of Endodontics 19, no. 11 (1993): 541–544. 10.1016/S0099-2399(06)81282-3 8151240

[10] M. Lim , C. Jung , D. H. Shin , Y. B. Cho , and M. Song , “Calcium Silicate‐Based Root Canal Sealers: A Literature Review,” Restorative Dentistry and Endodontics 45, no. 3 (2020): e35, 10.5395/rde.2020.45.e35. PMC7431927 32839716

[11] J. Modaresi and H. Aghili , “Sealing Ability of a New Experimental “Cold Ceramic” Material Compared to Glass Ionomer,” Journal of Clinical Dentistry 17, no. 3 (2006): 64–66. 17022367

[12] S. Hasheminia , J. Modaresi , S. Nejad , F. Mahjour , and O. Dianat , “Comparing the Sealing Properties of Mineral Trioxide Aggregate and an Experimental Ceramic Based Root End Filling Material in Different Environments,” Indian Journal of Dental Research 24, no. 4 (2013): 474–477, 10.4103/0970-9290.118399. 24047841

[13] J. Modaresi and H. R. Hemati , “The Cold Ceramic Material,” Dental Research Journal(Isfahan) 15, no. 2 (2018): 85–88. PMC5858076 29576770

[14] J. Modaresi , M. Mehrabanian , R. Marincsák , and M. Afshar , “Retreatment of a Tooth With Calcified Canals Using Cold Ceramic: A Case Report,” Cureus 17, no. 8 (2025): e90769, 10.7759/cureus.90769. PMC12455265 40995243

[15] J. E. Kim , J. B. Cho , W. J. Yi , et al., “Accidental Overextension of Endodontic Filling Material in Patients With Neurologic Complications: A Retrospective Case Series,” Dento Maxillo Facial Radiology 45, no. 5 (2016): 20150394, 10.1259/dmfr.20150394. PMC5084700 26915406

[16] Y. V. R. Gomes , A. A. Tavares , R. C. Barbosa , et al., “Biological Responses to Biomaterials: A Review,” Brazilian Journal of Medical and Biological Research 58 (2025): e14599, 10.1590/1414-431X2025e14599. PMC12068782 40367018

[17] B. Gomes , E. Aveiro , and A. Kishen , “Irrigants and Irrigation Activation Systems in Endodontics,” Brazilian Dental Journal 34, no. 4 (2023): 1–33, 10.1590/0103-6440202305577. PMC10642269 37909632

[18] E. Çulha and F. Tunç , “Assessment of Extruded Root Canal Filling Materials in Single‐Rooted Teeth Using Cone Beam Computed Tomography,” European Journal of Therapeutics 29, no. 3 (2023): 518–525, 10.58600/eurjther1720.

[19] C. C. da Silva , A. J. A. de Vasconcelos , L. A. H. de Lima , et al., “Root Canal Filling Material in Periapical Lesions: A Polarized and Fluorescent Light Microscopy Case Series,” Saudi Endodontic Journal 13, no. 2 (2023): 197–203, 10.4103/sej.sej_188_22.

[20] N. P. Solanki , K. K. Venkappa , and N. C. Shah , “Biocompatibility and Sealing Ability of Mineral Trioxide Aggregate and Biodentine as Root‐End Filling Material: A Systematic Review,” Journal of Conservative Dentistry 21, no. 1 (2018): 10–15, 10.4103/jcd.Jcd_45_17. PMC5852924 29628640

[21] A. Chamani , M. Forghani , and G. Asadi , “Cold Ceramic for Repairing Root Perforations: A Case Report,” Clinical Case Reports 13, no. 2 (2025): e70182, 10.1002/ccr3.70182. PMC11821458 39949583

[22] S. Khedmat , P. Sarraf , E. Seyedjafari , P. Sanaei‐Rad , and F. Noori , “Comparative Evaluation of the Effect of Cold Ceramic and MTA‐Angelus on Cell Viability, Attachment and Differentiation of Dental Pulp Stem Cells and Periodontal Ligament Fibroblasts: An In Vitro Study,” BMC Oral Health 21, no. 1 (2021): 628, 10.1186/s12903-021-01979-1. PMC8650362 34876089

[23] L. Demiriz and E. H. Bodrumlu , “Retrospective Evaluation of Healing of Periapical Lesions After Unintentional Extrusion of Mineral Trioxide Aggregate,” Journal of Applied Biomaterials & Functional Materials 15, no. 4 (2017): e382–e386, 10.5301/jabfm.5000359. 28525679

[24] A. Roshani , S. A. H. Baraz , and I. Shiezadeh , “Cold Ceramic in Endodontics Treatments: A Review,” Journal of Craniomaxillofacial Research 11, no. 3 (2024): 146–154.

